# Sodium-Glucose Cotransporter 2 (SGLT2) Inhibitor- and Metformin-Associated Euglycemic Diabetic Ketoacidosis and Lactic Acidosis Leading to Refractory Acute Kidney Injury: Successful Management With Hemodialysis

**DOI:** 10.7759/cureus.80989

**Published:** 2025-03-22

**Authors:** Norihito Yoshida, Tatsuki Tanaka, Yusuke Suzuki, Hiromasa Sakurai, Sadamu Takahashi, Mai Hitaka, Shingo Ishii, Jumpei Okuda, Keisuke Yamazaki, Yasushi Ohashi

**Affiliations:** 1 Nephrology, Toho University Sakura Medical Center, Sakura, JPN

**Keywords:** aki, dialysis care, high anion gap metabolic acidosis, metformin, sglt2 eudka

## Abstract

Euglycemic diabetic ketoacidosis (EuDKA) is a rare but serious complication of sodium-glucose cotransporter 2 (SGLT2) inhibitors, characterized by metabolic acidosis with near-normal blood glucose levels, making it challenging to diagnose. This case report describes a 49-year-old male with stage 4 chronic kidney disease (CKD) and type 2 diabetes mellitus (T2DM) on an SGLT2 inhibitor and metformin who developed EuDKA and lactic acidosis following an upper respiratory infection. Despite discontinuation of the medications and intravenous fluid resuscitation, severe metabolic acidosis persisted. Early initiation of hemodialysis led to rapid correction of acidemia, hyperkalemia, and improvement in renal function. This case highlights the importance of early recognition of SGLT2 inhibitor-associated EuDKA, particularly when complicated by lactic acidosis, and emphasizes the potential role of hemodialysis in managing refractory metabolic acidosis to prevent further clinical deterioration, even with modest hyperglycemia.

## Introduction

Euglycemic diabetic ketoacidosis (EuDKA) is a serious but often under-recognized complication of sodium-glucose cotransporter 2 (SGLT2) inhibitors. Unlike classic diabetic ketoacidosis, EuDKA occurs without marked hyperglycemia, which can lead to delayed diagnosis and treatment [1]. In patients with chronic kidney disease (CKD), concurrent metabolic disturbances, including lactic acidosis, further complicate management [2]. Here, we present a case of refractory EuDKA and lactic acidosis in a patient with stage 4 CKD taking an SGLT2 inhibitor and metformin, where hemodialysis was required to correct severe metabolic derangement. This case highlights the critical need for a high index of suspicion for EuDKA in patients taking SGLT2 inhibitors, even with only modest hyperglycemia, and underscores the importance of prompt intervention, including consideration of early hemodialysis, when standard therapies for metabolic acidosis fail.

## Case presentation

A 49-year-old male with a history of type 2 diabetes mellitus (T2DM), stage 4 chronic kidney disease (CKD) (baseline creatinine: 2.5 mg/dL, estimated glomerular filtration rate (eGFR): 23 mL/min/1.73 m²), pulmonary tuberculosis, pontine hemorrhage, diabetic retinopathy, and cerebral infarction, presented with a five-day history of flu-like symptoms and generalized fatigue. Four days prior to admission, he visited the emergency department with worsening symptoms and decreased oral intake despite a course of amoxicillin (AMPC) prescribed for a suspected upper respiratory infection. He had continued his regular medications, including dapagliflozin (an SGLT2 inhibitor) and metformin, without following a sick-day management protocol.

On admission, his vital signs were stable (temperature, 36.8°C; blood pressure, 115/73 mmHg; heart rate, 88 bpm (regular); respiratory rate, 24 breaths/min; SpO_2_, 99% on room air). Physical examination revealed a ketotic odor, dry tongue, and axillary dryness, suggesting dehydration and possible ketoacidosis. He denied urinary symptoms and abdominal pain and exhibited no skin rash, hemoptysis, or lower extremity edema. His primary complaints were related to upper respiratory tract symptoms.

Laboratory findings on admission (Table 1) revealed acute kidney injury (AKI) superimposed on CKD, with elevated serum creatinine and white blood cell count. He also had metabolic acidosis with an increased anion gap. Key findings included elevated serum lactate and blood ketones, with negative urine ketones. Urine sodium was low, suggesting a pre-renal etiology. The elevated spot urine protein and positive urine occult blood could be attributed to urine concentration secondary to reduced urine output, especially since the urine sediment was unremarkable and there was no evidence of hemolysis or rhabdomyolysis (normal lactate dehydrogenase (LDH) levels). Further investigations to rule out other causes of AKI and acidosis (Tables 2-4), including immune-mediated glomerulonephritis (specifically rapidly progressive glomerulonephritis (RPGN)) and endocrine dysfunction, were unremarkable, with negative antineutrophil cytoplasmic antibody (ANCA), antinuclear antibody (ANA), and anti-glomerular basement membrane (GBM) antibodies. Blood cultures were negative.

**Table 1:** Laboratory findings on admission. RBC: red blood cell count; Hb: hemoglobin; Ht: hematocrit; WBC: white blood cell count; platelet: platelet count; BUN: blood urea nitrogen; Cr: creatinine; eGFR: estimated glomerular filtration rate; uric acid: uric acid; TP: total protein; Alb: albumin; AST: aspartate aminotransferase; ALT: alanine aminotransferase; γ-GTP: gamma-glutamyl transferase; LDH: lactate dehydrogenase; blood glu: blood glucose; HbA1c: hemoglobin A1c; Na: sodium; K: potassium; Cl: chloride; Ca: calcium; P: phosphorus; CRP: C-reactive protein; Fe: iron; TIBC: total iron binding capacity; total ketone: total ketone bodies; Acetoacet: acetoacetate; 3-Hydroxy: 3-hydroxybutyric acid; EOS: eosinophils, LY: lymphocytes, MONO: monocytes, NE: neutrophils, NGSP: National Glycohemoglobin Standardization Program.

Parameter	Result and unit	Reference range
RBC	3.66 × 10⁶/µL	4.5-5.9 × 10⁶/µL
Hb	10.6 g/dL	13.5-17.5 g/dL
Ht	33.60%	38-50%
WBC	11,610/µL (NE 74%, LY 15.3%, MONO 6.4%, EOS 4.3%)	4,000-10,000/µL
Platelet	28.1 × 10⁴/µL	15-40 × 10⁴/µL
BUN	61.3 mg/dL	8-20 mg/dL
Cr	8.75 mg/dL	0.61-1.04 mg/dL
eGFR	6 mL/min/1.73m²	>60 mL/min/1.73m²
Uric acid	7.8 mg/dL	3.4-7.0 mg/dL
TP	7.5 g/dL	6.7-8.3 g/dL
Alb	3.5 g/dL	3.8-5.2 g/dL
AST	14 IU/L	10-40 IU/L
ALT	18 IU/L	5-45 IU/L
γ-GTP	19 IU/L	<80 IU/L
LDH	187 IU/L	124-222 IU/L
Blood glucose	103 mg/dL	73-109 mg/dL
HbA1c	7.1% (NGSP)	<6.5%
Na	136 mEq/L	135-145 mEq/L
K	5.3 mEq/L	3.5-5.0 mEq/L
Cl	101 mEq/L	98-108 mEq/L
Ca	8.3 mg/dL	8.4-10.4 mg/dL
P	6.5 mg/dL	2.5-4.5 mg/dL
CRP	4.4 mg/dL	<0.3 mg/dL
Fe	53 μg/dL	60-180 μg/dL
TIBC	250 μg/dL	250-450 μg/dL
Ferritin	112.1 ng/mL	30-300 ng/mL
Total ketone	166 µmol/L	<160 µmol/L
Acetoacetate	67.4 µmol/L	<40 µmol/L
3-Hydroxybutyrate	98.4 µmol/L	<60 µmol/L

**Table 2:** Blood gas analysis (venous, room air). pH: potential hydrogen, PaCO_2_: partial pressure of carbon dioxide, HCO₃⁻: bicarbonate, BE: base excess, lactate: blood lactate concentration.

Parameter	Result and unit	Reference range
pH	7.251	7.35-7.45
PaCO_2_	40 mmHg	35-45 mmHg
HCO_3_⁻	17.2 mmol/L	22-26 mmol/L
BE	-9.4 mmol/L	-3.0-3.0 mmol/L
Lactate	3.51 mmol/L	0.5-2.2 mmol/L
Anion gap	19.1 mEq/L	8-12 mEq/L

**Table 3:** Urinalysis on admission. Urine RBC (HPF): red blood cells per high-power field in urine sediment, urine WBC (HPF): white blood cells per high-power field in urine sediment, urine Na: urinary sodium, urine Cr: urinary creatinine.

Parameter	Result and unit	Reference range
Urine glucose	2+	Negative
Urine protein	4+	Negative
Urine occult blood	2+	Negative
Urine ketone bodies	Negative	Negative
Urine RBC (HPF)	<1	0-4
Urine WBC (HPF)	<1	0-4
Urine Na	<10 mEq/L	40-220 mEq/L
Urine Cr	4.09 mg/dL	0.6-1.5 mg/dL
Urine protein	20.78 g/gCr	<0.15 g/gCr

**Table 4:** Immunological and endocrine laboratory findings on admission. IgG: immunoglobulin G, IgA: immunoglobulin A, IgM: immunoglobulin M, C3: complement component 3, C4: complement component 4, CH50: total complement activity, ASO: antistreptolysin O, ANA: antinuclear antibody, Anti-dsDNA: anti-double-stranded DNA antibody, MPO-ANCA: myeloperoxidase-antineutrophil cytoplasmic antibody, PR3-ANCA: proteinase 3-antineutrophil cytoplasmic antibody, Anti-GBM Ab: anti-glomerular basement membrane antibody, TSH: thyroid-stimulating hormone, FT3: free triiodothyronine, FT4: free thyroxine, ACTH: adrenocorticotropic hormone, Anti-GAD Ab: anti-glutamic acid decarboxylase antibody.

Parameter	Result and unit	Reference range
IgG	1,257 mg/dL	700-1,600 mg/dL
IgA	251 mg/dL	70-400 mg/dL
IgM	90 mg/dL	40-230 mg/dL
C3	118 mg/dL	80-180 mg/dL
C4	30 mg/dL	10-40 mg/dL
CH50	52 IU/mL	40-60 IU/mL
ASO	49 IU/mL	<200 IU/mL
ANA	<40 (Speckled)	<40
Anti-dsDNA IgG	2.8 IU/mL	<5 IU/mL
MPO-ANCA	<0.2 U/mL	<3.5 U/mL
PR3-ANCA	<0.6 U/mL	<3.5 U/mL
Anti-GBM Ab	<1.5 U/mL	<1 U/mL
TSH	0.87 µIU/mL	0.5-5.0 µIU/mL
FT3	1.86 pg/mL	2.3-4.2 pg/mL
FT4	0.83 ng/dL	0.8-1.9 ng/dL
ACTH	9.3 pg/mL	7.2-63.3 pg/mL
Cortisol	10.0 µg/dL	3.7-19.4 µg/dL
Anti-GAD Ab	<5.0 U/mL	<5.0 U/mL

**Figure 1:**
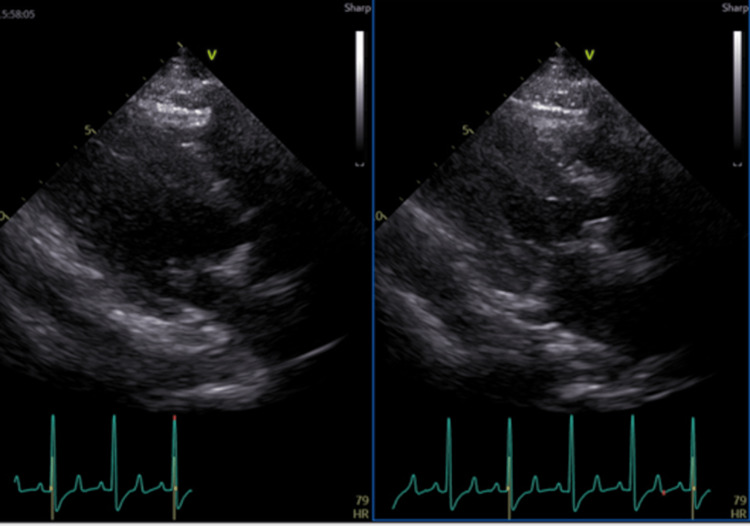
Transthoracic echocardiographic findings. TTE revealing a preserved LVEF of 79%, with no evidence of valvular dysfunction or pericardial effusion, indicative of normal cardiac function. LVEF: left ventricular ejection fraction, TTE: transthoracic echocardiography.

**Figure 2:**
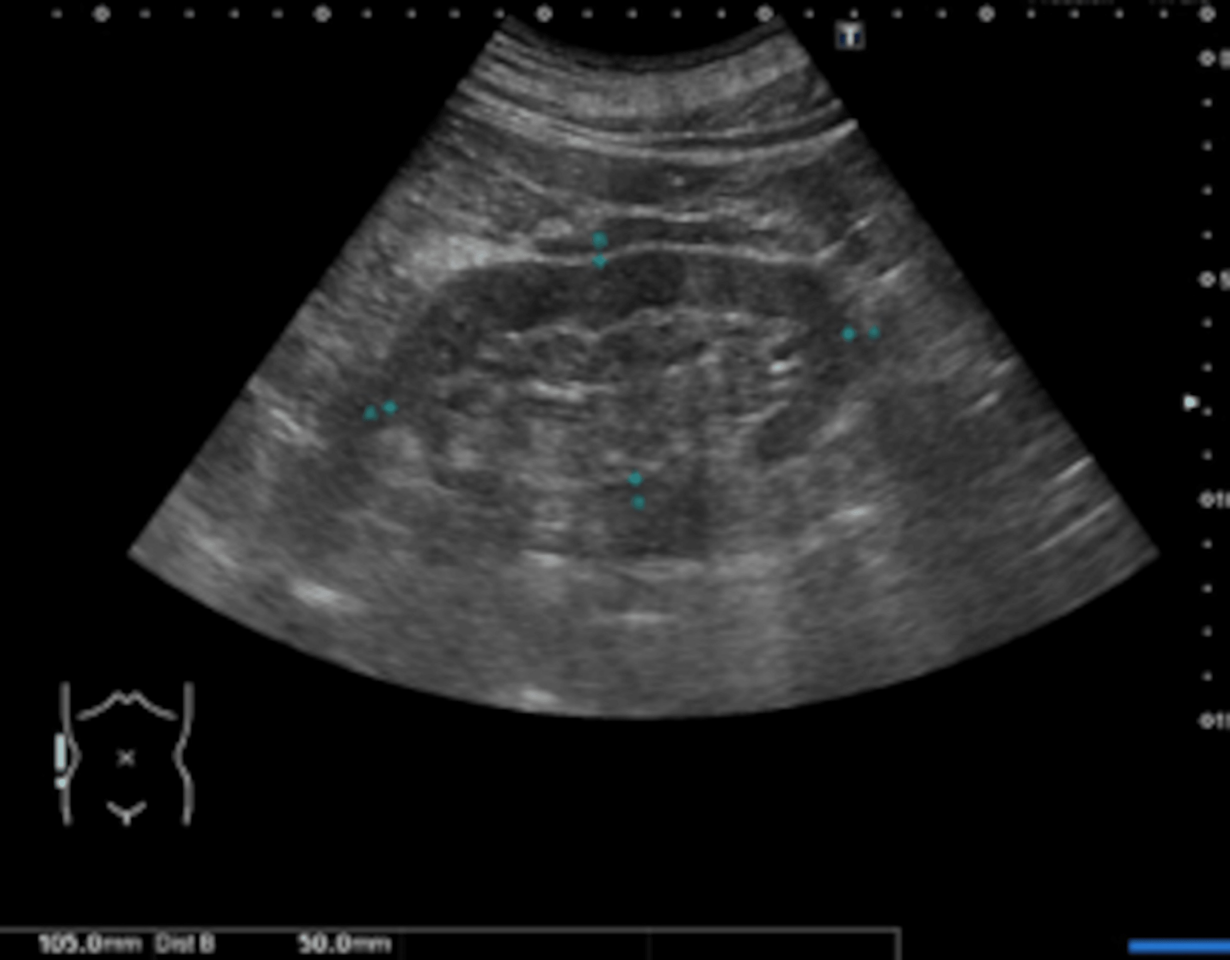
Right renal ultrasound findings. Renal ultrasound demonstrates irregular morphology with mild atrophy of the right kidney, which measured 105 mm in length and 50 mm in width, with no evidence of hydronephrosis.

**Figure 3:**
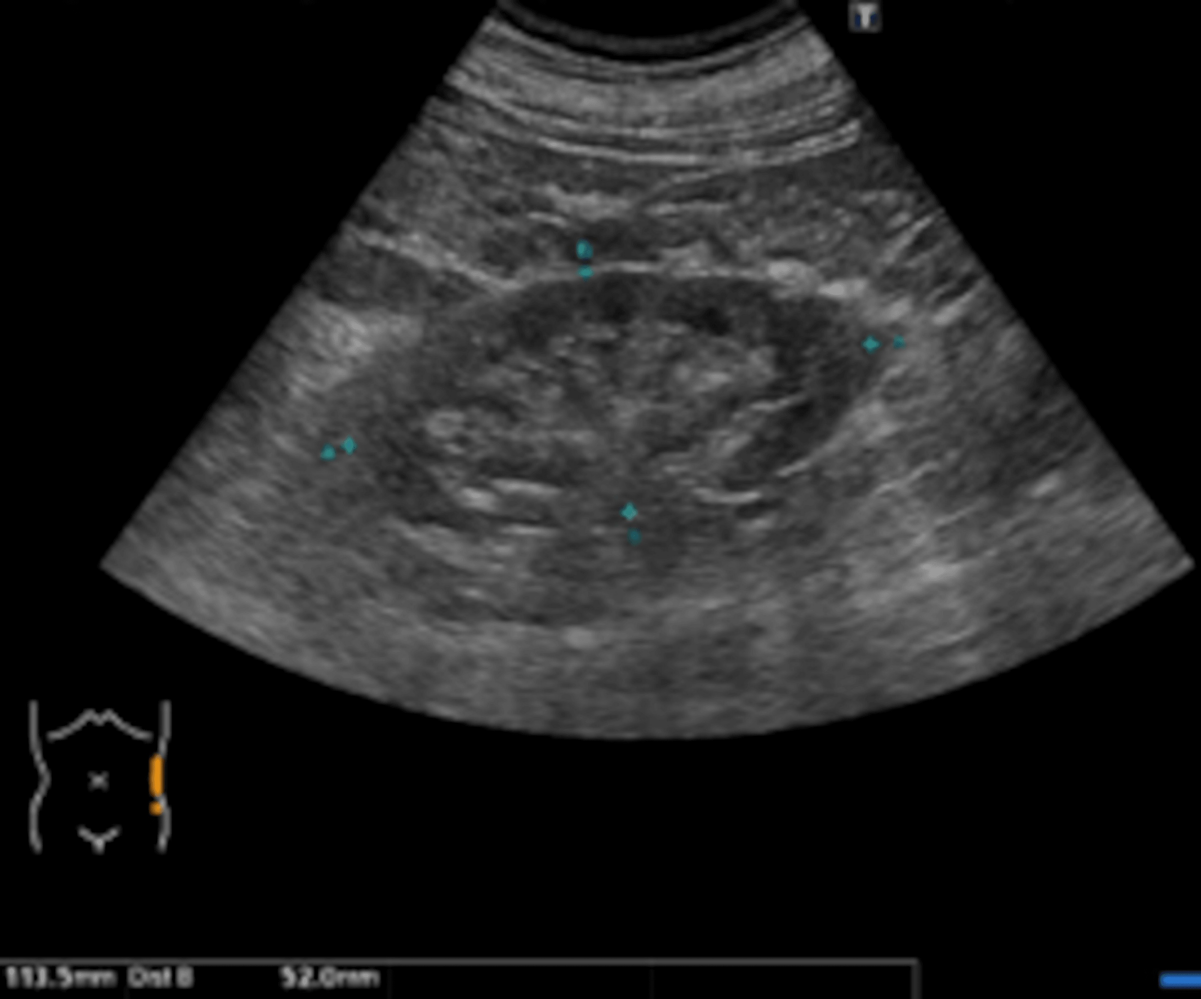
Left renal ultrasound findings. Renal ultrasound demonstrates irregular morphology with mild atrophy of the left kidney, which measured 113.5 mm in length and 52 mm in width, with no evidence of hydronephrosis.

**Figure 4:**
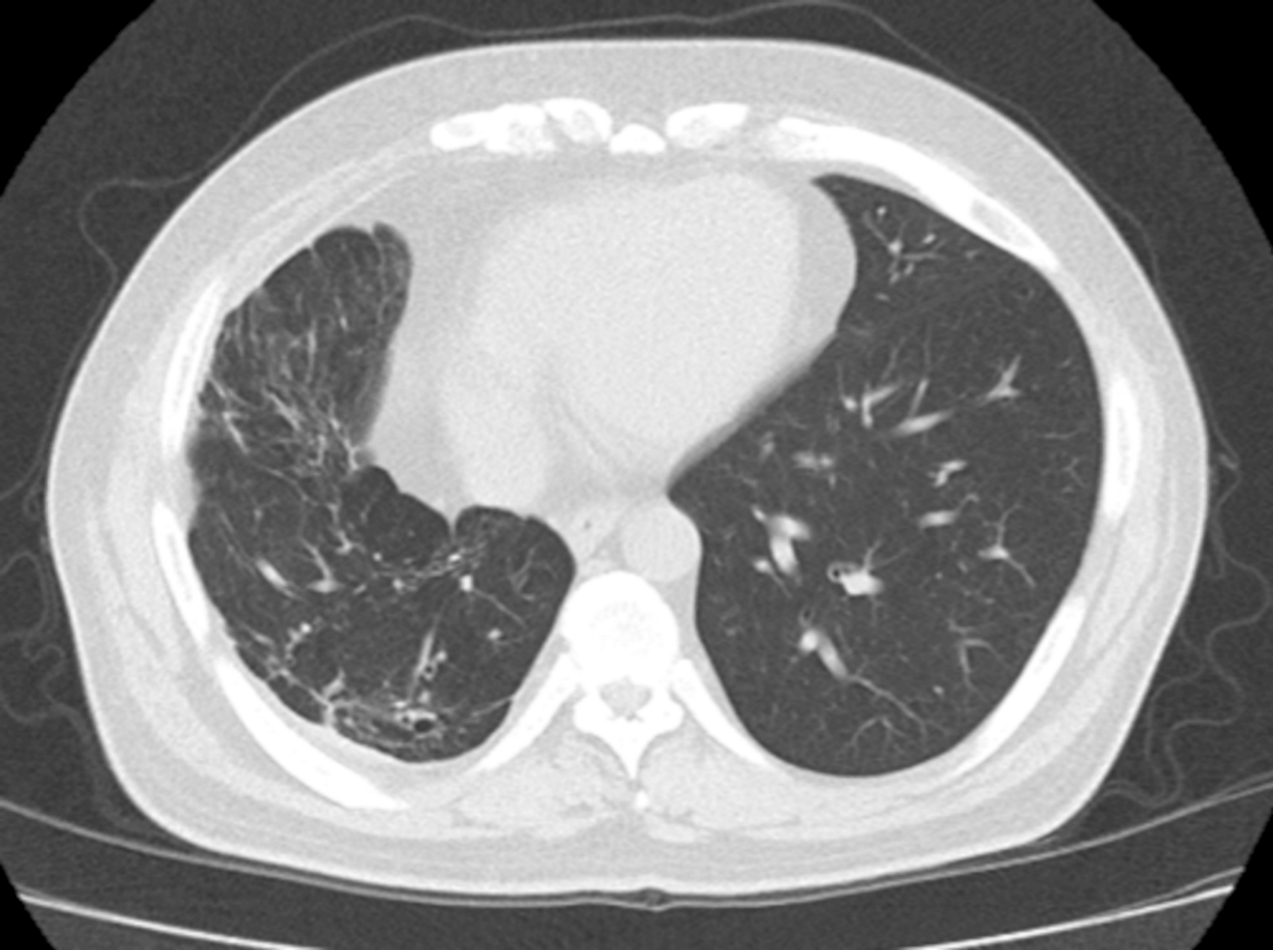
Chest CT findings. Non-contrast chest CT showing no radiographic evidence of pneumonia or pleural effusion.

Imaging studies (Figures 1-4) showed no evidence of acute cardiopulmonary or renal abnormalities other than findings consistent with his known history (prior pulmonary tuberculosis, CKD). Specifically, the ECG and echocardiogram ruled out acute heart failure, renal ultrasound excluded post-renal obstruction by showing no hydronephrosis, and chest CT showed no evidence of pneumonia or other infection that could contribute to the acidosis.

Given the presence of dehydration, metabolic acidosis, elevated blood ketones, elevated lactate, and ongoing SGLT2 inhibitor and metformin use, a diagnosis of EuDKA complicated by volume depletion and metformin-associated lactic acidosis (MALA) was suspected. SGLT2 inhibitor and metformin were discontinued immediately. Given the patient's dehydration and ongoing metabolic acidosis, he was started on intravenous fluid therapy, consisting of Ringer's lactate solution (1 L) with added sodium bicarbonate (40 mL of 8.4% solution, providing approximately 40 mEq of bicarbonate) administered continuously over 24 hours.

By hospital day two, he remained unresponsive to conservative therapy, with persistent oliguria (urine output: 150 mL/day) and worsening metabolic acidosis. Due to the refractory acidosis and the need for toxin clearance, hemodialysis was initiated via a temporary blood access catheter. The dialysis regimen was as follows: DVS-100NX dialysis device (Nikkiso Co., Ltd. Tokyo, Japan), FB-90 dialyzer (Nipro Corporation, Osaka, Japan), Kindaly AF-4 dialysate, dialysate flow rate of 300 mL/min, blood flow rate of 100 mL/min, temperature of 36°C, initial heparin bolus of 500 units followed by continuous infusion of 500 units/hour, duration of four hours, and ultrafiltration rate of 0.1 L/hour.

Following the initial hemodialysis session, the patient's urine output increased significantly, and he was able to resume oral intake. On hospital day three, a blood gas analysis was performed, showing improvement in his acidosis (Lac: 1.86 mmol/L, pH: 7.358, HCO_3_^-^: 25.3 mmol/L, BE: -3.9 mmol/L). However, given the severity of his initial presentation and the risk of rebound acidosis, a second hemodialysis session was performed with the same parameters. By hospital day four, the patient's metabolic acidosis and renal function had significantly improved, and with no evidence of recurrence of acidosis, hemodialysis was discontinued.

**Figure 5:**
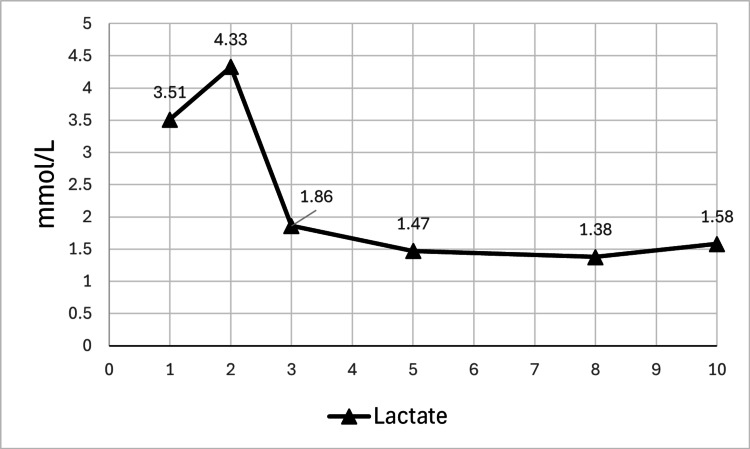
Changes in serum lactate levels after initiation of hemodialysis and discontinuation of SGLT2 inhibitor and metformin. Changes in serum lactate levels (mmol/L) after initiation of hemodialysis and discontinuation of SGLT2 inhibitor and metformin. The patient's serum lactate levels were monitored serially via venous blood gas analysis. Hemodialysis was initiated on day two and performed again on day three. SGLT2 inhibitor and metformin were discontinued on admission. SGLT2: sodium-glucose cotransporter 2.

Lactate levels showed a decreasing trend following dialysis and medication discontinuation (Figure 5). A follow-up blood ketone measurement on day eight showed a total ketone body level of 115 μmol/L (acetoacetate: 48.6 μmol/L, 3-hydroxybutyrate: 66.3 μmol/L). His subsequent recovery was uneventful, with no recurrence of acidosis or further deterioration in renal function. His urine output remained stable, and oral intake was well maintained. 

**Figure 6:**
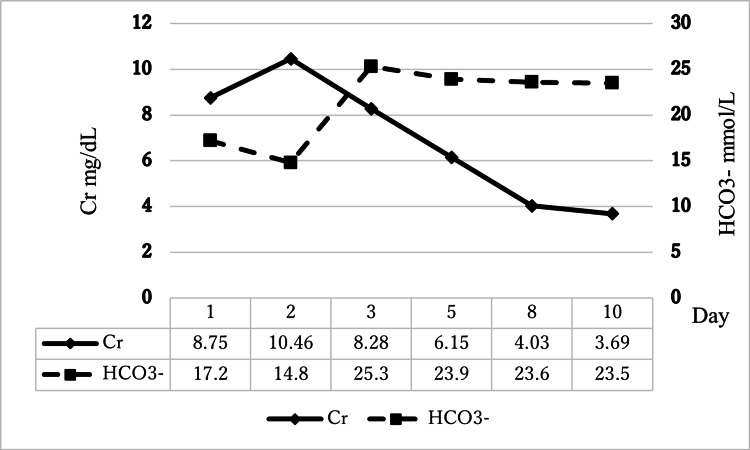
Clinical course of serum creatinine and bicarbonate levels during hospitalization. The patient’s renal function and acid-base status during hospitalization are shown in figure. On admission (day one), serum creatinine (Cr) was 8.75 mg/dL, and bicarbonate (HCO₃⁻) was 17.2 mmol/L, indicating AKI with metabolic acidosis. Despite fluid resuscitation, Cr increased to 10.46 mg/dL on day two, while HCO₃⁻ dropped to 14.8 mmol/L, prompting hemodialysis on days two and three. Following hemodialysis, Cr levels progressively declined to 3.69 mg/dL by day 10, and HCO₃⁻ stabilized above 23 mmol/L, suggesting effective metabolic correction and renal recovery.

The patient was discharged on hospital day 10 on dulaglutide and mitiglinide for glycemic control. He had been followed up regularly by a nephrologist in the outpatient setting, and his creatinine and eGFR have remained at his baseline levels, with no recurrence of acidosis (Figure 6). This case report describes a 49-year-old male with type 2 diabetes and stage 4 CKD who presented with EuDKA and suspected MALA, requiring hemodialysis for refractory metabolic acidosis despite standard therapy.

## Discussion

This case highlights the diagnostic and therapeutic challenges of EuDKA complicated by lactic acidosis in a patient with CKD. Unlike typical DKA, EuDKA occurs with near-normal blood glucose levels, which can lead to delayed diagnosis and treatment [1]. While SGLT2 inhibitor-associated EuDKA has been increasingly reported [2], few cases have documented concurrent lactic acidosis and required hemodialysis for refractory metabolic acidosis, making this case particularly noteworthy.

EuDKA is a well-recognized complication of SGLT2 inhibitors. The mechanism involves increased urinary glucose excretion, leading to lower plasma glucose and reduced insulin secretion. The resulting relative insulin deficiency stimulates glucagon release, promoting hepatic ketogenesis. Concurrently, lipolysis increases, providing free fatty acids for β-oxidation and further ketone body production [3,4]. Dehydration, often exacerbated by the osmotic diuretic effect of SGLT2 inhibitors, and intercurrent illness, such as the upper respiratory infection in this case, can compromise renal function, impairing clearance of lactate, and thus worsening the metabolic acidosis [5].

In this patient, the combination of pre-existing stage 4 CKD, dehydration, and continued use of both an SGLT2 inhibitor and metformin likely contributed to the development of severe metabolic acidosis. While the clinical presentation and laboratory findings were consistent with EuDKA, the elevated lactate level raised concern for concurrent lactic acidosis. Although the patient's lactate did not meet the strict criteria for MALA [6], metformin's renal excretion and the patient's AKI likely contributed to its accumulation and potential contribution to lactic acidosis. We considered both type A and type B lactic acidosis. Type A lactic acidosis was considered possible due to the patient's hypovolemia, resulting from decreased oral intake, the osmotic diuretic effect of the SGLT2 inhibitor, and potential renal hypoperfusion, as suggested by the low urine sodium. Type B lactic acidosis was also considered due to the patient's use of metformin. Although metformin levels were not measured, the patient's AKI likely impaired metformin clearance, potentially contributing to lactic acidosis. Other causes of lactic acidosis were considered less likely. Sepsis was considered less likely given the absence of fever, hypotension, or other signs of systemic infection and the negative blood cultures. Tissue ischemia was also considered less likely, given the absence of significant peripheral vascular disease, signs of mesenteric ischemia, or altered mental status. D-lactic acidosis was ruled out due to the absence of relevant surgical history, such as short bowel syndrome.

Standard therapy for EuDKA and lactic acidosis includes fluid resuscitation and discontinuation of the offending agents [3,6]. However, despite these measures, our patient's metabolic acidosis remained severe and refractory, necessitating hemodialysis. While some EuDKA cases resolve with conservative management, this case highlights that severe, refractory metabolic acidosis may require more aggressive intervention [7,8]. Hemodialysis effectively corrects acidemia and removes lactate and, potentially, metformin, although the latter's large volume of distribution may lead to rebound [6,9]. Importantly, the rapid improvement in acid-base status and renal function following hemodialysis suggests its crucial role in this patient's recovery.

Several limitations in this case are warrant consideration. Although a preceding viral upper respiratory infection was suspected, other potential infectious etiologies cannot be entirely ruled out. Despite negative blood cultures, prior antibiotic administration may have influenced the results. Additionally, given that no urine or arterial blood gas analysis was performed during the initial emergency department visit, it remains unclear whether the patient was already acidotic at presentation. Furthermore, the sequence of events, whether lactic acidosis or ketoacidosis developed first or whether both conditions progressed concurrently, remains uncertain. The adequacy of sodium bicarbonate administration for acidosis correction is another unresolved factor. Although hemodynamic compromise and circulatory failure were likely major contributors to lactic acidosis, the extent to which metformin played a role in its pathogenesis remains indeterminate. Furthermore, although the clinical course and other laboratory data did not strongly suggest it, we cannot definitively rule out the possibility of rapidly progressive glomerulonephritis (RPGN) superimposed on diabetic nephropathy, as a kidney biopsy was not performed. While the spot urine protein-to-creatinine ratio on admission was significantly elevated (20.78 g/gCr), this could be attributed to urine concentration secondary to reduced urine output. Importantly, the patient's baseline urinary protein excretion ranged from 1 to 3 g/gCr, and his proteinuria remained in this range after the resolution of the acute episode, making a superimposed RPGN less likely. In addition, while iron studies ruled out iron deficiency anemia, we did not measure vitamin B12, folate, or other micronutrient levels, nor did we perform a further evaluation for monoclonal gammopathies (e.g., serum-free light chain assays, protein electrophoresis, immunofixation). However, the patient lacked findings suggestive of multiple myeloma, such as hypercalcemia or pathological fractures, and his immunoglobulins were not suppressed.

## Conclusions

Clinicians should maintain a high index of suspicion for EuDKA in patients on SGLT2 inhibitors, particularly when unexplained metabolic acidosis occurs, even with only modest hyperglycemia. This case demonstrates that lactic acidosis can coexist with EuDKA, further complicating management. When metabolic acidosis persists despite intravenous fluids and drug discontinuation, early hemodialysis should be considered to prevent further deterioration. Further large-scale studies are needed to evaluate the optimal treatment strategies, including the role and timing of hemodialysis, in SGLT2 inhibitor-associated metabolic complications.
